# DECIPHER-PRAD: an advanced fragmentomics-based cell-free DNA assay for prostate cancer early detection

**DOI:** 10.1186/s12964-025-02522-3

**Published:** 2025-11-29

**Authors:** Shun Zhang, Guanchen Zhu, Linfeng Xu, Qing Zhang, Xuefeng Qiu, Hua Bao, Min Wu, Xiaotian Zhao, Tao Ding, Fufeng Wang, Shuang Chang, Yang Shao, Junlong Zhuang, Hongqian Guo

**Affiliations:** 1 Department of Urology, Nanjing Drum Tower Hospital Clinical College of Nanjing Medical University, Nanjing, 210008 China; 2 Department of Urology, Nanjing Drum Tower Hospital, Affiliated Hospital of Medical School, Nanjing University, Nanjing, 210008 China; 3 Geneseeq Research Institute, Nanjing Geneseeq Technology Inc., Nanjing, 210032 China; 4 School of Public Health, Nanjing Medical University, Nanjing, Jiangsu 211166 China

**Keywords:** Early detection, Prostate cancer, Fragmentomics, Cell-free DNA

## Abstract

Early detection of prostate cancer is limited by the poor specificity of prostate-specific antigen (PSA)-based screening. Cell-free DNA (cfDNA) fragmentomics offers a promising non-invasive approach to improve screening accuracy and risk stratification. In this study, we enrolled 106 prostate cancer patients and 114 high-risk non-cancer individuals to develop a cfDNA fragmentomics-based screening assay using plasma whole-genome sequencing. Two fragmentomic features—copy number variation and fragment size profile—were incorporated into machine learning models for training and evaluated in an independent validation cohort of 83 cancer patients and 76 non-cancer individuals. The fragmentomics-based model achieved an area under the curve (AUC) of 0.933 in the training cohort (66.0% sensitivity at 95.6% specificity; 51.9% sensitivity at 98.2% specificity) with good calibration (slope: 0.957; intercept: 0.001), and maintained strong performance in the validation cohort (AUC: 0.887; 57.8% sensitivity at 92.1% specificity), showing rising predictive probabilities and sensitivity across advancing stages (Stage I–IV: 27.3% to 77.8%). Importantly, the model performed well in the PSA grey zone (4–10 ng/mL) with an AUC of 0.865 (69.0% sensitivity at 81.8% specificity). When integrated with total PSA levels, the combined algorithm achieved an AUC of 0.915 in the validation cohort and improved sensitivity at 98% specificity (Stage I–IV: 30.0% to 87.5%). These findings support the clinical potential of our cfDNA fragmentomic assay, particularly when combined with PSA, as a highly accurate and non-invasive tool for early prostate cancer detection.

**Supplementary Information:**

The online version contains supplementary material available at 10.1186/s12964-025-02522-3.

## Introduction

Prostate cancer remains the most prevalent malignancy affecting men worldwide, ranking as the second most frequently diagnosed cancer and the fifth leading cause of cancer-related mortality among males globally [1]. According to the World Health Organization’s 2022 estimates, approximately 1.4 million new cases were reported, demonstrating significant geographic variations linked to socioeconomic disparities, dietary patterns, and healthcare access influencing screening rates [2, 3]. While pathologically characterized as indolent in many cases, the disease poses significant clinical challenges when progressing to aggressive, particularly in high-risk cases defined by Gleason grading (≥ 8) exhibiting rapid tumor doubling times and disorganized architecture [4, 5]. This malignancy demonstrates strong age-related penetrance, with incidence rates rising from 1.8% in men 60–69 year to 9.0% in men 70 year and older [6]. Current screening relies primarily on prostate-specific antigen (PSA) testing with a 4 ng/mL threshold; however, this approach suffers from suboptimal specificity leading to overdiagnosis [7–9].

Emerging liquid biopsy technologies exploiting cell-free DNA (cfDNA) have brought promise for addressing these limitations. cfDNA derived from apoptotic or necrotic tumor cells, carries cancer-specific genomic and epigenomic aberrations detectable in plasma [10]. While mutation-centric approaches using targeted panel sequencing show promise [11], their performance remains constrained by technical limitations, such as detecting germline mutation alone [12]. Recent studies suggest that fragmentomics analysis of whole-genome sequencing (WGS)-derived cfDNA fragmentation patterns may overcome the potential barriers [13, 14]. Recent studies integrating a variety of fragmentomic features have revealed advantages of stacked prediction model developed by machine learning algorithms in cancer early detection. For instance, a weighted diagnostic model based on genome-wide 5-hydroxymethylcytosine, nucleosome footprint, 5′ end motif, and fragmentation profiles of cfDNA was employed for early detection of hepatocellular carcinoma in cirrhotic patients [15]. Sensitive stacked ensemble models combining multiple machine learning algorithms for early detection have also been developed in lung cancer [16], colorectal cancer [17], and breast cancer [18].

In this study, we developed a novel fragmentomics-based liquid biopsy assay leveraging WGS data for early prostate cancer detection. Using machine learning algorithms trained on curated fragmentomic features, we established a classifier and validated it in an independent cohort. Moreover, the diagnostic accuracy was further enhanced by integrating PSA measurements, creating a multimodal detection platform. This work highlights fragmentomics’ potential as a non-invasive high-sensitivity screening tool with translational implications for prostate cancer screening.

## Methods

### Participant enrollment and study cohorts

This study was approved by the Institutional Review Board (IRB) of the Nanjing Drum Tower Hospital, Affiliated Hospital of Medical School of Nanjing University (No. 2023–006-01), and adhered to the Declaration of Helsinki. All patients provided written informed consent prior to sample collection.

**Fig. 1:**
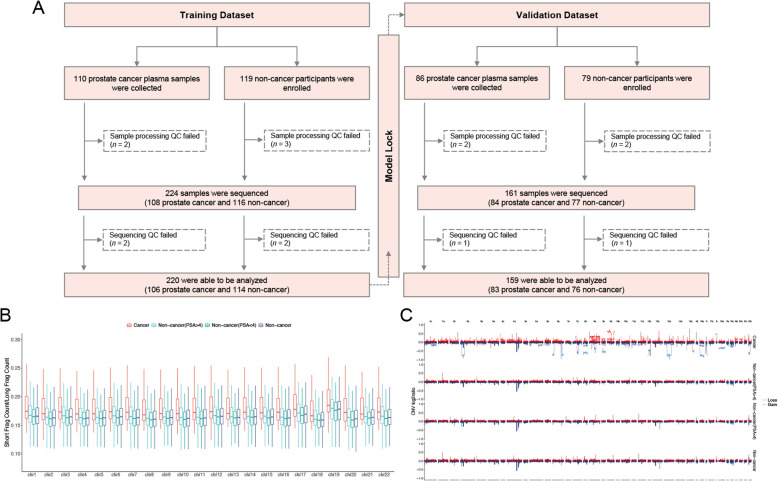
Flowchart of study design and fragmentomic feature comparison. **A** Flowchart illustrating the study design, cohort composition, and sample sizes. **B** Comparison of the ratio of short to long cfDNA fragments within 5 Mb bins across chromosomes 1–22 between prostate cancer and non-cancer samples in the training cohort. **C** Comparison of CNV log2 ratios across 1 Mb bins spanning chromosomes 1–22 between prostate cancer and non-cancer samples in the training cohort

We prospectively enrolled 110 patients diagnosed with prostate cancer and 119 non-cancer high-risk males between April 2024 and December 2024 at the Nanjing Drum Tower Hospital, Affiliated Hospital of Medical School of Nanjing University for assay development via machine learning algorithms and performance evaluation by leave-one-out cross-validation (LOOCV). An independent validation cohort included 86 patients with prostate cancer and 79 non-cancer high-risk males were collected in a separate time frame between January 2025 and April 2025 at the same site. Prostate cancer patients meeting the following inclusion criteria were included in this study: (1) histologically diagnosed prostate cancer; (2) without history of other cancer diagnosis; (3) available plasma liquid biopsy at initial diagnosis of prostate cancer. Non-cancer high-risk male participants who were healthy volunteers fulfilling the following inclusion criteria were incorporated in this study: (1) without history of any cancer diagnosis at enrollment; (2) available plasma liquid biopsy obtained during routine physical checks at enrollment; (3) having not been diagnosed with any cancer by the end of this study. Participants whose plasma samples failed either sample processing or sequencing quality control were excluded (Fig. 1A).

### cfDNA extraction and whole-genome sequencing

Venous blood samples were obtained during routine physical checks for non-cancer participant when they were recruited in this study. Prostate cancer patients’ venous blood samples were collected upon patients’ admission, which was within 3 weeks after initial diagnosis and prior to subsequent surgical or systemic treatment. A same standard protocol was applied to the collection, processing, and sequencing of blood samples to minimize the variations. A full blood sample (8–10 mL) from each participant was collected using an EDTA tube at the Nanjing Drum Tower Hospital, Affiliated Hospital of Medical School of Nanjing University. To separate plasma, samples were centrifuged at 1800 × g for 10 min at room temperature within 2 h, followed by secondary centrifugation at 18,000 × g to remove residual cellular debris within 6 h. All sample were stored at −80℃ until shipped with dry ice to a College of American Pathologists, Clinical Laboratory Improvement Amendments, and ISO 15189-qualified central laboratory of Nanjing Geneseeq Technology Inc. (Nanjing, China). The following sequencing steps were performed within 24 h of cfDNA extraction from plasma using QIAamp Circulating Nucleic Acid Kit (Qiagen) following manufacturer’s instructions. cfDNA quality and concentration were evaluated using the Qubit dsDNA HS Assay Kit (Thermo Fisher Scientific). 5–10 ng plasma cfDNA of each sample was subjected to polymerase chain reaction (PCR)-free WGS library construction using the KAPA HyperPrep Kit (Roche). A total of nine cfDNA samples were excluded owing to < 5 ng (failure rate, 2.28%). A maximum of 10 ng cfDNA was used to construct sequencing library if extracted cfDNA exceeded 10 ng.

Prepared libraries were subjected to paired-end sequencing on the MGI T7 platform. Initial trimming of FASTQ files was performed by Trimmomatic as a part of the quality control [19]. PCR duplicates were removed using the Picard toolkit (http://broadinstitute.github.io/picard/), and qualified reads were aligned to the human reference genome (GRCh37/UCSC hg19) by BWA [19]. The sequencing quality control index consisted of Q30, GC content, proportion of reads aligned to reference genome, median insert, and mean depth. A total of six samples failed sequencing quality control. The sample operating and sequencing team were blinded to the sample status (cancer/non-cancer) throughout the entire process.

### Bioinformatic analysis

Two types of fragmentomic features of chromosomes 1–22 from WGS data were extracted for assay development, including copy number variation (CNV), and fragmentation size coverage (FSC). CNV refers to the gains or losses of chromosomal region > 1 kb, which is associate with cancer when the amplifications or deletions occur on oncogenes or tumor suppressor genes; FSC captures fragment size characteristic of cfDNA. CNV features were extracted from BAM files using ichorCNA developed by Wan et al. with adaption [20]. The genome was tiled into a total of 2475 1 Mb disjoint bins with respect to the reference genome. After correcting the coverage depth of each bin by GC content and comparing against the software baseline, the log2 ratio of each bin was calculated by ichorCNA. The FSC extraction procedure was adapted from S. Cristiano et al.’s method [14], dividing the genome into 541 non-overlapping 5 Mb bins. Coverages of short fragments (100–150 bp) and long fragments (151–220 bp) in each bin were calculated and standardized. Therefore, a total of 2475 CNV log2 ratios and 1082 FSC scores across chromosomes 1–22 were generated.

### Assay development by machine learning algorithm

The input data for CNV and FSC features had 2475 and 1082 variables, respectively, with each column considered as a variable prior to feature selection. Per-fold feature selection was performed independently within each fold of LOOCV in the training cohort. In each LOOCV iteration, the CNV log2 ratios of each 1 Mb bin were compared between cancer samples and non-cancer samples by student's t-test to identify statistically significant difference with *p* < 0.01, and similarly, the FSC scores of each 5 Mb bin were tested to select bins with significant difference reaching* p* < 0.01 between cancer and non-cancer samples. Gradient Boosting Machine (GBM) algorithms were used to build prediction models based on retained CNV features and FSC features separately in the training cohort to generate CNV and FSC scores ranging from 0 to 1, which represented the predictive probabilities of being a prostate cancer sample via CNV or FSC fragmentomic features. A fragmentomic score between 0 and 1 aggregating the CNV and FSC scores was generated by constructing a Generalized Linear Model (GLM). The entire training procedure was repeated for each LOOCV iteration to ensure unbiased performance estimation. For final model development after LOOCV evaluation, CNV log2 ratios of 380 bins and FSC scores of 158 bins selected using student’s t-tests on the entire training cohort, were used to train models on the full dataset. The training involving GBM and GLM algorithms were conducted using the H2O AutoML [21]. The hyperparameter optimization in H2O AutoML was performed using a random grid search approach. Hyperparameters of GBM algorithms were randomly sampled from predefined distributions to explore diverse configurations. The GLM algorithm required a nuanced approach: H2O AutoML conducted an internal grid search, systematically evaluating combinations of alpha and lambda. The integrated algorithm including the CNV score, FSC score, and total PSA level was established by eXtreme Gradient Boosting (XGBoost) using the *xgboost* package in R [22] based on the training cohort (eval_metric = "logloss", max_depth = 3, gamma = 1, alpha = 0.5, subsample = 0.75, colsample_bytree = 0.7, eta = 0.01, nrounds = 1000, early_stopping_rounds = 20). After fixing the parameters of each individual and integrated prediction model, the threshold value was determined at targeted 98.0% specificity of a subcohort including non-cancer individuals with PSA ≥ 4 ng/mL from the training cohort.

### Permutation analysis

All feature values were rescaled into z-scores to ensure comparability, with PSA concentrations log-transformed prior to standardization. For each permutation, the values of one feature were randomly shuffled across samples while the other two features remained unchanged. The shuffled dataset was used to recalculate the AUC. This procedure was repeated 2000 times, and the mean AUC across all permutations was recorded as the null distribution. Feature importance was quantified as:

$$\triangle\mathrm{AUC}\;=\;\mathrm{Mean}\;{\mathrm{AUC}}_{\mathrm{original}-\;}\mathrm{Mean}\;{\mathrm{AUC}}_{\mathrm{after}\;\mathrm{shuffling}}$$

### F1 score

The F1 score was used as a metric to comprehensively evaluate the performance of different assays. The calculation was done as below:

$${\text{F}}_{1}=2 \times \frac{\text{Precision }\times \text{ Sensitivity}}{\text{Precision}+\text{Sensitivity}}$$

### Statistical analysis

Descriptive analyses were performed on clinical characteristics of enrolled participants. Fisher’s exact/Chi-squared tests were used to compare the frequencies of categorical variables. Student's t-test or Wilcoxon rank-sum tests were used to test the difference in continuous variables. Data were analyzed using R software (version 4.2.2). Receiver operating characteristic (ROC) curves and area under the curve (AUC) were generated to assess the discrimination using the *pROC* package. Sensitivity, specificity, positive predictive value, negative predictive value, and accuracy calculations were performed using the *epiR* package. Calibration and SHAP plots were outputted using the *rms* and *shapviz* packages, respectively. A two-tailed *p* < 0.05 was considered statistically significant unless otherwise stated. The bootstrap test was employed to estimate the distribution of the F1 score using 2000 bootstrap iterations.

## Results

### Participant characteristics

A total of 220 participants were enrolled in the training cohort, including 106 patients with prostate cancer and 114 non-cancer individuals (Fig. 1A). The independent validation cohort included 159 participants, consisting of 83 prostate cancer patients and 76 non-cancer individuals. The clinical characteristics of two cohorts are summarized in Table S1. Most prostate cancer patients were diagnosed with stage II diseases (training, 52.8%; validation, 45.8%) or 3 + 4 Gleason grades identified through tissue biopsies (training, 36.8%; validation, 41.0%). Relatively high PSA levels were observed in cancer patients in both the training (mean, 19.34 ng/mL) and validation (mean, 37.48 ng/mL) cohorts. The average PSA levels of non-cancer participants of the training and validation cohorts were 3.43 and 3.61 ng/mL, with individuals having PSA < 4 ng/mL accounting for 52.6% and 60.5% respectively. 56.1% and 49.1% of non-cancer individuals in the training cohort reported prostatic enlargement and calcification, respectively. Similarly, the most prevalent non-malignant prostatic abnormalities in the validation cohort were enlargement (48.7%) and calcification (48.7%).

### Fragmentomic features and fragmentomic-based screening assay developed by machine learning algorithms

FSC and CNV fragmentomic features were obviously distinct between prostate cancer patients and non-cancer participants. A trend of larger FSC scores was observed in prostate cancer patients in the training cohort, representing more common short fragments in cancer samples than in non-cancer samples, while there appeared to be some non-cancer samples with PSA ≥ 4 ng/mL which exhibited increased FSC scores when compared to those with PSA < 4 ng/mL (Fig. 1B and Figure S1). Significant chromosomal arm CNV gain with log2 ratio > 0 was observed in 2p, 8q, 9q, 11q, etc., and CNV loss identified by log2 ratio < 0 was detected in 2p, 6q, 13q, etc. (Fig. 1C). Similar CNV fragmentomic features were observed between non-cancer samples with PSA ≥ 4 and < 4 ng/mL.

**Fig. 2:**
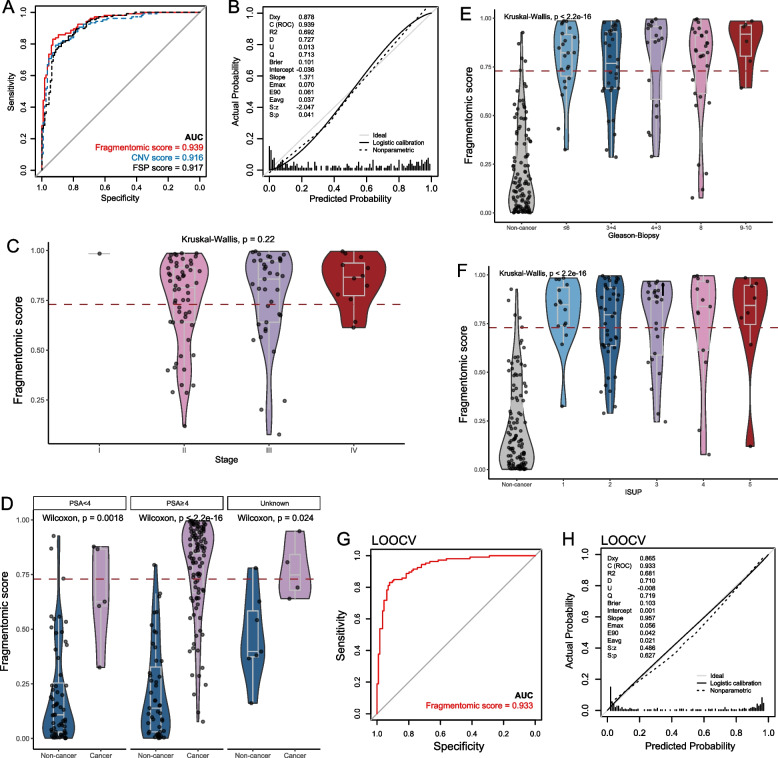
The performance of the fragmentomics-based assay in the training cohort. **A**, **B** Discrimination and calibration curves of the fragmentomics-based assay in the training cohort. **C** Comparison of fragmentomic scores across prostate cancer samples with different disease stages. **D** Comparison of fragmentomic scores between cancer and non-cancer samples stratified by PSA levels. **E**, **F** Comparison of fragmentomic scores between non-cancer and cancer samples across different Gleason or ISUP grades. **G**, **H** Discrimination and calibration of the assay using LOOCV to mitigate overfitting

Given considerable differences in fragmentomic features between prostate cancer and non-cancer samples, a stacked fragmentomic model combining FSC and CNV features was developed based on the training cohort by the GBM algorithm. The stacked fragmentomic model achieved an AUC of 0.939 when distinguishing prostate cancer samples from non-cancer samples in the training cohort, with a trend of improved performance compared to using FSC (AUC, 0.917) or CNV (AUC, 0.916) features alone (Fig. 2A). We performed calibration analysis to evaluate how well the predicted probabilities of prostate cancer aligned with observed diagnosis, thereby reflecting the reliability of the risk scores in real-world clinical application. The stacked model exhibited good calibration, with an intercept of −0.036 indicating minimal systematic bias in predictions and a slope of 1.371 suggesting generally reliable predictions with only slight overfitting (Fig. 2B). To minimize the false positive events among healthy, non-cancer individuals, which is particularly important in a population-wide screening context, while preserving sensitivity for detecting prostate cancer in high-risk groups, the fragmentomic score threshold of 0.7294 was selected. This cutoff was chosen to achieve a specificity of 98.0% among individuals with PSA ≥ 4 ng/mL, while maintaining a sensitivity of 68.0%. Within the entire training cohort (sensitivity, 66.0%; specificity, 95.6%), a trend of increased fragmentomic predictive scores was observed with higher disease stage (Fig. 2C), and the sensitivity of stage I–IV were 100.0% (1/1), 62.5% (35/56), 64.9% (24/37), 83.3% (10/12), respectively. Notably, two of five prostate cancer patients with PSA < 4 ng/mL were successfully identified by the fragmentomic assay, showing significantly higher predictive scores (*p* < 0.01, Fig. 2D). The predictive scores of subgroups with various Gleason grades defined via biopsy and The International Society of Urological Pathology (ISUP) grades defined via surgery were presented in Fig. 2E and F, respectively. Intriguingly, differences in predictive scores were hardly observed across cancer samples with various grades.

The performance was then evaluated by LOOCV to reduce the overestimation due overfitting, yielding a slightly lower AUC of 0.933 (Fig. 2G), sensitivity of 45.8% at specificity of 98.2%, significantly higher predictive scores in cancer samples than non-cancer samples (Figure S2), and good calibration with intercept of 0.001 and slope of 0.957 (Fig. 2H).

### Outstanding discrimination in the PSA grey area

**Fig. 3:**
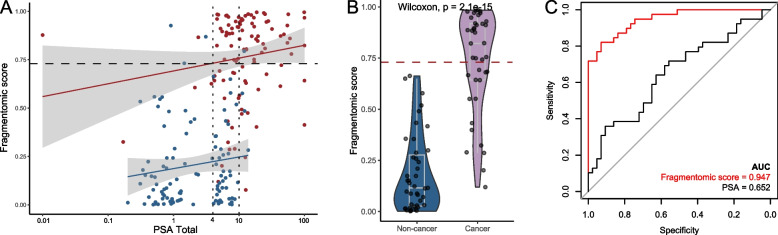
The performance of the fragmentomics-based assay among males having PSA within the gray zone. **A** Scatter plot of fragmentomic scores and PSA levels for each sample. The red dots represent cancer samples, and the blue dots represent non-cancer samples. **B** Among individuals with PSA levels between 4–10 ng/mL, cancer samples exhibited significantly higher fragmentomic scores than non-cancer samples. **C** The fragmentomics-based assay demonstrated superior discriminative performance compared to PSA alone in the gray zone subgroup

Given the relatively poor specificity of PSA screening using a cut-off of 4 ng/mL, it is hard to distinguish cancer from non-cancer samples having PSA level within a grey area between 4 and 10 ng/mL. Figure 3A displays the fragmentomic score and matched PSA level of each sample. Approximately half of samples whose PSA levels were within the grey area were from non-cancer participants exhibiting fragmentomic predictive scores lower than the threshold. Moreover, prostate cancer samples had significantly higher predictive scores than non-cancer samples (*p* < 0.01), and all non-cancer samples showed predictive scores below the threshold of 0.7294 (61.5% sensitivity at 100.0% specificity, Fig. 3B). The fragmentomic-based assay exhibited outstanding discrimination achieving an AUC of 0.947 compared to that of PSA (AUC, 0.652; Fig. 3C).

### Performance assessment in an independent validation cohort

**Fig. 4:**
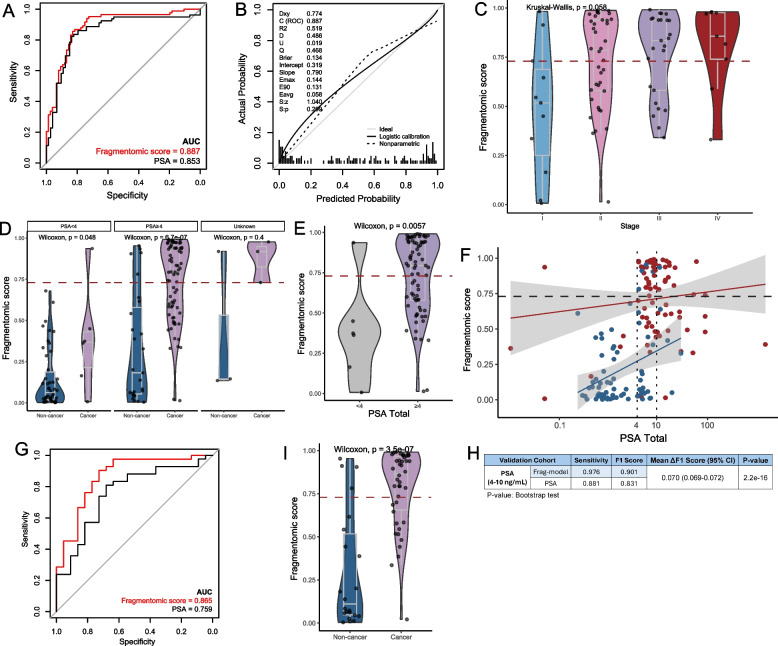
The performance of the fragmentomics-based assay in the independent validation cohort. **A**, **B** Discrimination and calibration curves of the fragmentomics-based assay in the validation cohort. **C** Comparison of fragmentomic scores across cancer samples with varying disease stages. **D** Comparison of fragmentomic scores between cancer and non-cancer samples stratified by PSA levels. **E** Comparison of fragmentomic scores between cancer samples with low vs. high PSA levels. **F** Scatter plot of fragmentomic scores and PSA levels for each sample in the validation cohort. The red dots represent cancer samples, and the blue dots represent non-cancer samples. **G**-**H** The assay outperformed PSA alone in distinguishing cancer from non-cancer cases in the gray zone. **I** Within the gray zone, cancer samples showed significantly higher fragmentomic scores than non-cancer samples

In the validation cohort, the AUC of the fragmentomic-based assay was 0.887 (57.8% sensitivity at 92.1% specificity), slightly higher than that of PSA (0.853, Fig. 4A). In terms of calibration, the intercept and slope were 0.319 and 0.790, respectively (Fig. 4B). A trend of elevated fragmentomic predictive scores (*p* = 0.058) and sensitivity (I, 27.3%; II, 55.3%; III, 68.0%; IV, 77.8%) was observed with the increase of disease stages (Fig. 4C). The predictive scores of samples with various Gleason and ISUP grades were presented in Figure S3. Similarly to the training cohort, the fragmentomic predictive scores of cancer samples were higher than those of non-cancer samples regardless of the PSA level (Fig. 4D). Our data also revealed that prostate cancer samples with ≥ 4 ng/mL PSA had greater predictive scores than those with < 4 ng/mL PSA (*p* = 0.006, Fig. 4E). The assay performance among samples having PSA values within the grey area remained excellent (Fig. 4F), with an AUC of 0.865 (69.0% sensitivity at 81.8% specificity), which was better than PSA (AUC, 0.759; Fig. 4G). The fragmentomic-based model achieved an F1 score of 0.901, significantly outperforming PSA, which had an F1 score of 0.831 (*p* < 0.001, Fig. 4H). As expected, there was a significantly elevated predictive scores in cancer samples compared to non-cancer samples (*p* < 0.001, Fig. 4I).

### Development and evaluation of an integrated algorithm combining fragmentomic features and PSA

**Fig. 5:**
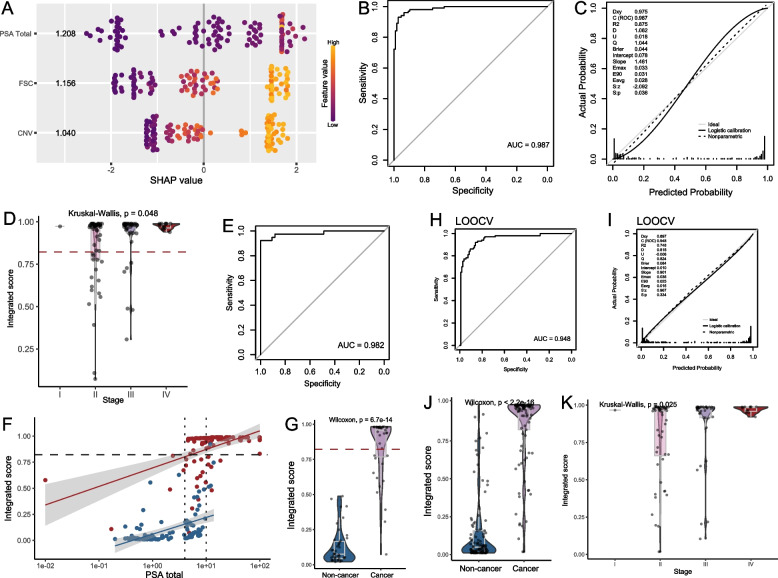
The performance of the integrated model involving PSA in the training cohort. **A** SHAP summary plots of the integrated model incorporating PSA levels, FSC scores, and CNV scores. Features were ranked on y-axis by their mean absolute SHAP values, indicating overall contribution to model predictions (PSA Total = 1.208, FSC = 1.156, CNV = 1.040). Each dot represented an individual sample, with its position on x-axis showing the SHAP value which indicates its impact on prediction. Dot color reflected the corresponding feature value, as shown in the color bar (purple = low, yellow = high). **B**, **C** Discrimination and calibration of the integrated model in the training cohort. **D** Comparison of integrated scores across cancer samples at different disease stages. **E** ROC curve for the integrated model applied to samples with PSA in the gray zone. **F** Scatter plot of integrated scores and PSA levels in the training cohort. The red dots represent cancer samples, and the blue dots represent non-cancer samples. **G** Cancer samples showed significantly higher fragmentomic scores than non-cancer samples within the PSA gray zone. **H**, **I** Discrimination and calibration of the integrated model under LOOCV. **J** Under LOOCV, cancer samples had significantly higher integrated scores than non-cancer samples. **K** Comparison of integrated scores across cancer samples with different disease stages under LOOCV

We next aimed to develop an integrated model that combined cfDNA fragmentomic profiles with the protein biomarker. The model was built using XGBoost algorithm, incorporating total PSA levels, FSC scores, and CNV scores. To evaluate the contribution of each feature to prediction outcomes, we examined the SHAP values and performed permutation analysis. As shown in the SHAP summary plot (Fig. 5A), all three features contribute similarly while PSA emerged as the most influential feature, although its effect on model predictions varied, with both low and high values driving predictions in different directions. FSC and CNV demonstrate more consistent patterns, where higher feature values were positively associated with cancer prediction. The permutation analysis showed consistent results as all features contribute similarly. Disruption of PSA scores resulted in 6.031% decrease in AUC, while disruption of FSC (∆AUC = 5.878%) and CNV (∆AUC = 5.468%) also led to declines in AUC (Figure S4).

**Fig. 6:**
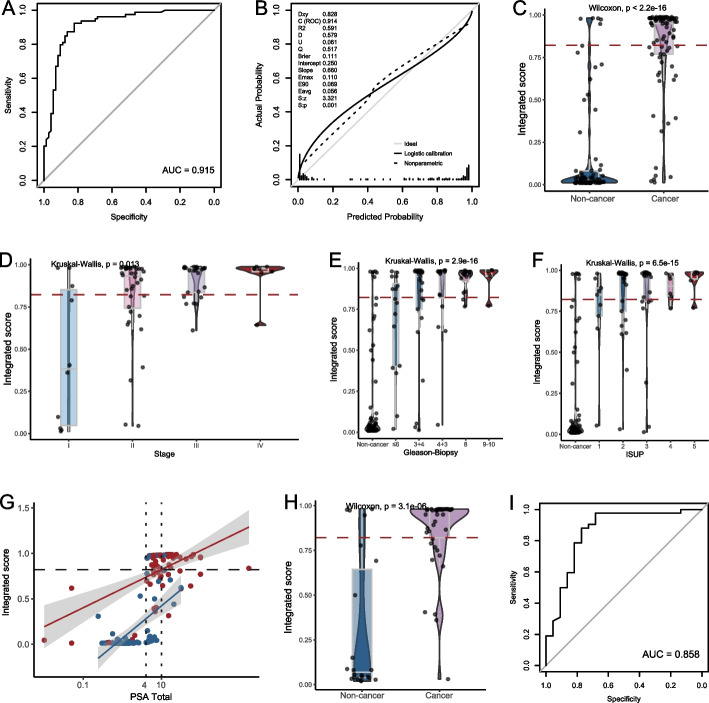
The performance of the integrated model involving PSA in the independent validation cohort. **A**, **B** Discrimination and calibration of the integrated model in the validation cohort. **C** Cancer samples exhibited significantly higher integrated scores than non-cancer samples. **D** Comparison of integrated scores across prostate cancer samples with varying disease stages. **E**, **F** Comparison of integrated scores between non-cancer and cancer samples grouped by Gleason or ISUP grade. **G** Scatter plot of integrated scores and PSA levels in the validation cohort. The red dots represent cancer samples, and the blue dots represent non-cancer samples. **H** Within the PSA gray zone, cancer samples had significantly higher fragmentomic scores than non-cancer samples. **I** ROC curve of the integrated model for individuals with PSA levels in the gray zone

The discrimination and calibration are shown in Fig. 5B and C, achieving an AUC of 0.987, intercept of 0.078, and slope of 1.461. Significantly elevated integrated scores from stage I to IV diseases were observed (*p* = 0.048, Fig. 5D). The predictive scores of samples with various Gleason and ISUP grades were presented in Figure S5. The integrated algorithm also showed good performance in the grey area (AUC, 0.982; Fig. 5E) and significantly higher integrated scores in cancer samples than non-cancer samples (*p* < 0.001, Fig. 5F, G). The results of LOOCV demonstrated similar discrimination (AUC, 0.948; 68.3% sensitivity at 98.1% specificity; Fig. 5H) and calibration (intercept, 0.010; slope, 0.901; Fig. 5I). In the validation cohort, the AUC of the integrated algorithm was 0.915, with 67.1% sensitivity at 93.2% specificity (Fig. 6A). Good calibration (Fig. 6B) and significantly difference in predictive scores (*p* < 0.001, Fig. 6C) between cancer and non-cancer samples were observed. As expected, there was a trend of increased predictive scores from stage I to stage IV diseases (Fig. 6D) or with the increase of grades (Fig. 6E, F). Its discrimination within the grey area remained a high-level performance (AUC, 0.858) even though four non-cancer samples having predictive scores over the threshold (Fig. 6G-I).

## Discussion

In this prospective study, we developed and validated a cfDNA-based fragmentomic assay incorporating both copy number variation (CNV) and fragmentation size coverage (FSC) features, demonstrating robust performance in distinguishing prostate cancer from non-cancer high-risk individuals. Importantly, the assay showed excellent discrimination even among participants with PSA levels within the diagnostic grey area (4–10 ng/mL), a long-standing clinical challenge in prostate cancer screening.

Our findings highlight that cfDNA fragmentomic characteristics, such as increased short fragment representation and focal CNV gains/losses, are significantly altered in prostate cancer patients compared to non-cancer individuals. These alterations were effectively captured using genome-wide WGS at shallow coverage, enabling a non-invasive and scalable approach for early cancer detection. The combined FSC and CNV model achieved high AUC values in both the training (AUC, 0.933) and validation (AUC, 0.887) cohorts, with minimal evidence of overfitting as indicated by LOOCV. This supports the biological relevance and technical robustness of the fragmentomic signals captured.

Critically, the cfDNA fragmentomic-based assay markedly outperformed PSA in the 4–10 ng/mL grey zone, achieving an AUC of 0.947 in the training and 0.865 in the validation cohort, versus 0.652 and 0.759 for PSA, respectively. By integrating total PSA levels with our assay through an ensemble model, we achieved an AUC of 0.948 in the training cohort and 0.915 in the validation cohort. The integrated assay leverages the widespread availability and established clinical role of PSA while adding orthogonal biological information from cfDNA, resulting in a more informative and reliable screening tool for early prostate cancer detection.

Nonetheless, this study has limitations. Firstly, although our cohorts were prospectively collected and independently validated, the overall sample size remains modest, particularly in subgroups such as patients with PSA < 4 ng/mL or stage I disease. Larger multicenter studies are necessary to confirm generalizability and performance in more diverse populations. Secondly, although shallow WGS is more cost-effective than deep sequencing, implementing this assay at scale will require further cost–benefit analyses and potential optimization for clinical practicality.

In conclusion, our cfDNA fragmentomic assay, particularly when integrated with PSA, represents a promising non-invasive tool for prostate cancer screening. By addressing the diagnostic limitations of PSA alone, especially within the grey zone, this integrated approach holds potential for improving early detection and personalized risk stratification. Further validation in broader populations and prospective clinical trials will be essential for advancing this strategy toward clinical application.

## Supplementary Information

Supplementary Material 1.

Supplementary Material 2.

## Data Availability

The datasets used and/or analyzed during the current study are available from the corresponding author on reasonable request.
