# Supplementary figures and images for: DECIPHER-PRAD: an advanced fragmentomics-based cell-free DNA assay for prostate cancer early detection
Source: Cell Commun Signal. 2025 Nov 29;24:2. doi: 10.1186/s12964-025-02522-3 (PMC12765298; doi:10.1186/s12964-025-02522-3)

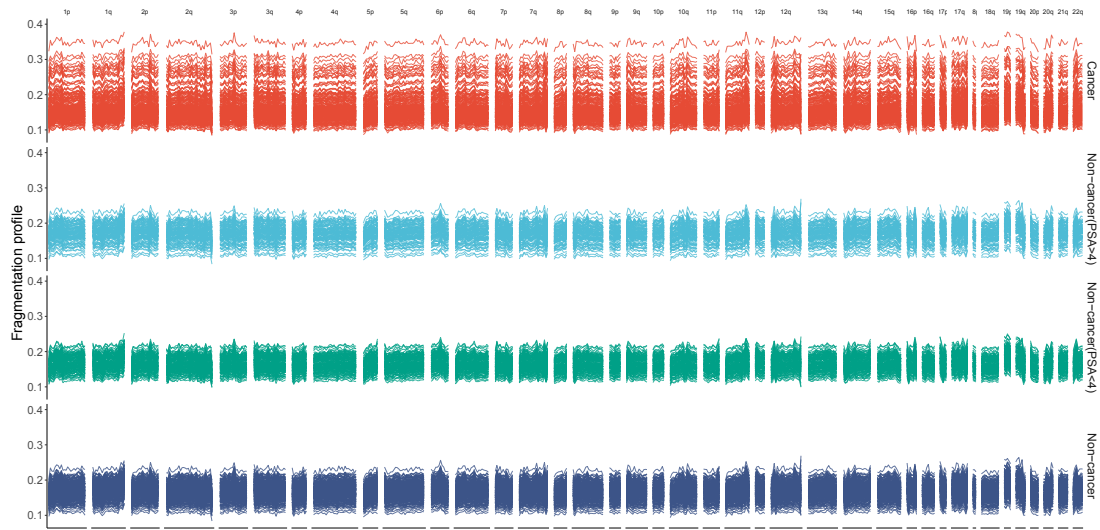

Supplement: Supplementary file 2 — Supplementary Material 2. [file 12964_2025_2522_MOESM2_ESM.zip › Figure s1.pdf]

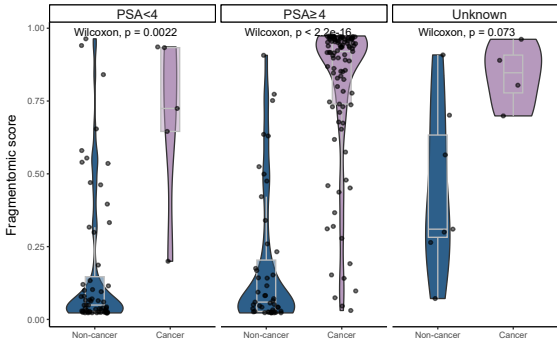

Supplement: Supplementary file 2 — Supplementary Material 2. [file 12964_2025_2522_MOESM2_ESM.zip › Figure s2.pdf]

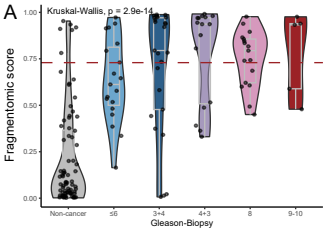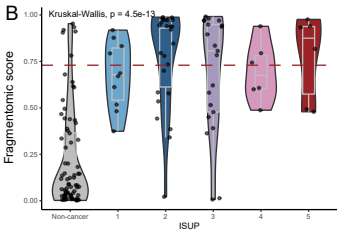

Supplement: Supplementary file 2 — Supplementary Material 2. [file 12964_2025_2522_MOESM2_ESM.zip › Figure s3.pdf]

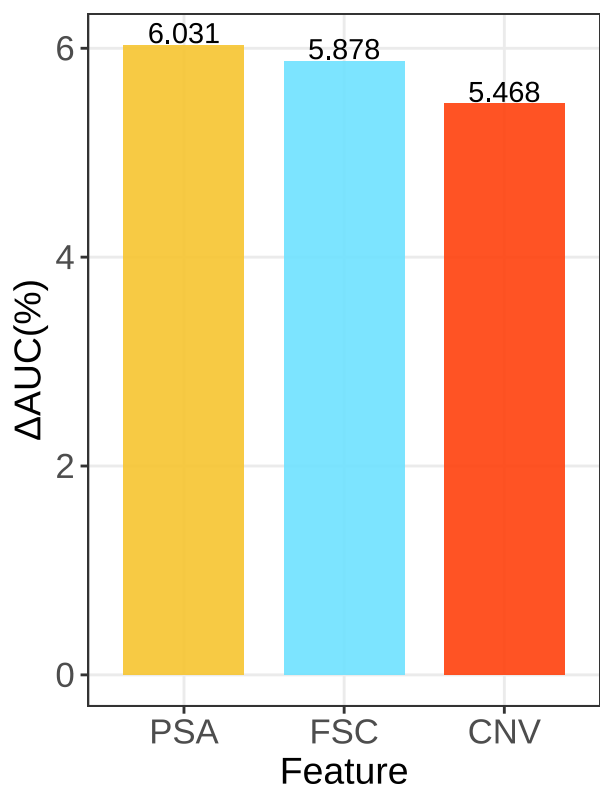

Supplement: Supplementary file 2 — Supplementary Material 2. [file 12964_2025_2522_MOESM2_ESM.zip › Figure S4.pdf]

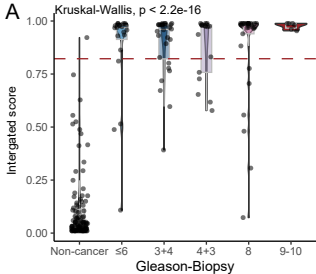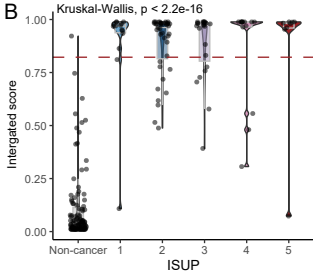

Supplement: Supplementary file 2 — Supplementary Material 2. [file 12964_2025_2522_MOESM2_ESM.zip › Figure s5.pdf]
